# Cardiorespiratory Alterations in a Newborn Ovine Model of Systemic Inflammation Induced by Lipopolysaccharide Injection

**DOI:** 10.3389/fphys.2020.00585

**Published:** 2020-06-17

**Authors:** Stéphanie Nault, Vincent Creuze, Sally Al-Omar, Annabelle Levasseur, Charlène Nadeau, Nathalie Samson, Roqaya Imane, Sophie Tremblay, Guy Carrault, Patrick Pladys, Jean-Paul Praud

**Affiliations:** ^1^Neonatal Respiratory Research Unit, Departments of Pediatrics and Pharmacology-Physiology, Université de Sherbrooke, Sherbrooke, QC, Canada; ^2^LIRMM, Univ Montpellier, CNRS, Montpellier, France; ^3^CHU Sainte-Justine Research Center, Departments of Neurosciences and Pediatrics, Faculty of Medicine, Université de Montréal, Montreal, QC, Canada; ^4^Inserm, LTSI – UMR 1099, CHU Rennes, Université Rennes 1, Rennes, France

**Keywords:** lipopolysaccharides, neonatal sepsis, cardiorespiratory control, heart-rate variability, respiratory-rate variability

## Abstract

Although it is well known that neonatal sepsis can induce important alterations in cardiorespiratory control, their detailed early features and the mechanisms involved remain poorly understood. As a first step in resolving this issue, the main goal of this study was to characterize these alterations more extensively by setting up a full-term newborn lamb model of systemic inflammation using lipopolysaccharide (LPS) injection. Two 6-h polysomnographic recordings were performed on two consecutive days on eight full-term lambs: the first after an IV saline injection (control condition, CTRL); the second, after an IV injection of 2.5 μg/kg *Escherichia coli* LPS 0127:B8 (LPS condition). Rectal temperature, locomotor activity, state of alertness, arterial blood gases, respiratory frequency and heart rate, mean arterial blood pressure, apneas and cardiac decelerations, and heart-rate and respiratory-rate variability (HRV and RRV) were assessed. LPS injection decreased locomotor activity (*p* = 0.03) and active wakefulness (*p* = 0.01) compared to the CTRL. In addition, LPS injection led to a biphasic increase in rectal temperature (*p* = 0.01 at ∼30 and 180 min) and in respiratory frequency and heart rate (*p* = 0.0005 and 0.005, respectively), and to an increase in cardiac decelerations (*p* = 0.05). An overall decrease in HRV and RRV was also observed. Interestingly, the novel analysis of the representations of the horizontal and vertical visibility network yielded the most statistically significant alterations in HRV structure, suggesting its potential clinical importance for providing an earlier diagnosis of neonatal bacterial sepsis. A second goal was to assess whether the reflexivity of the autonomic nervous system was altered after LPS injection by studying the cardiorespiratory components of the laryngeal and pulmonary chemoreflexes. No difference was found. Lastly, preliminary results provide proof of principle that brainstem inflammation (increased IL-8 and TNF-α mRNA expression) can be shown 6 h after LPS injection. In conclusion, this full-term lamb model of systemic inflammation reproduces several important aspects of neonatal bacterial sepsis and paves the way for studies in preterm lambs aiming to assess both the effect of prematurity and the central neural mechanisms of cardiorespiratory control alterations observed during neonatal sepsis.

## Introduction

Sepsis is defined as a systemic inflammation caused by a bacterial, viral, or fungal infection (Hotchkiss et al., 2016; Singer et al., 2016). Neonatal sepsis remains a major problem, especially in preterm infants with late-onset sepsis (LOS), in whom it causes substantial morbidity and mortality (Mayr et al., 2014; Shane et al., 2017). Early diagnosis of sepsis is challenging because the initial clinical signs are non-specific (Fanaroff et al., 1998). While recurrent apnea/bradycardias are frequently observed during LOS, more subtle alterations in heart rate (HR) and respiratory-rate variability (RRV) are often the first and sole manifestations detectable in the early phase of sepsis (Beuchée et al., 2009). The currently available monitoring of heart-rate variability (HRV) allows earlier diagnosis of LOS (Griffin et al., 2005; Bohanon et al., 2015), with a reported decrease in death rate of 20% (Fairchild, 2013). This approach is, however, hampered by low specificity, which limits its clinical usefulness. The use of HRV analysis methods with better diagnostic accuracy are needed to further improve the early LOS detection and prognosis (Coggins et al., 2016). Moreover, a better understanding of the altered cardiorespiratory control during neonatal sepsis is needed.

Two studies in rodents by a Swedish team concluded that the inflammatory response to systemic injection of lipopolysaccharides (LPS), taken as a model of bacterial sepsis, inhibits respiration (Olsson et al., 2003; Hofstetter et al., 2007). This team then found a correlation between the number of apneas and infected newborns (Siljehav et al., 2015). These results were, however, restricted to the alterations in respiratory frequency (f_R_) and apnea events; no information was provided on the altered HR and bradycardias. While a few other studies of neonatal sepsis in animal models—including lambs (Billiards et al., 2002; McClure et al., 2005; Hamilton et al., 2006; Feng et al., 2010), rodents (Olsson et al., 2003; McDonald et al., 2016a, b), and piglets (McDeigan et al., 2003)—revealed certain cardiorespiratory alterations, the duration of all these studies was limited to a few minutes and/or the time course of these alterations were not detailed. Overall, to our knowledge, available results have not fully characterized the cardiorespiratory-control alterations present in newborns in the early phase of sepsis. As a first step in our research program, the main goal of the present study was to set up a full-term newborn lamb model of systemic inflammation using LPS injection to characterize those alterations more extensively using continuous recordings of cardiac and respiratory activity. A second goal was to assess whether the reflexivity of the autonomic nervous system was altered after LPS injection by studying the cardiorespiratory components of the laryngeal and pulmonary chemoreflexes. Lastly, we aimed at providing preliminary results in a few additional lambs as proof of principle that brainstem inflammation may be involved in the alterations in cardiorespiratory control observed in our neonatal ovine model.

## Materials and Methods

The study was carried out in accordance with the recommendations of the Canadian Council on Animal Care. The study protocol was approved by the Ethics Committee for Animal Care and Experimentation of the Université de Sherbrooke (protocol #409-16). A total of 20 full-term, mixed-bred male lambs, aged 2–4 days, were included in the study.

### Chronic Instrumentation and Recording Equipment

Chronic surgical instrumentation was performed under local anesthesia with 2% lidocaine and conscious sedation with ketamine 5 mg/kg, preceded by anafen 3 mg/kg + atropine sulfate 0.1 mg/kg. Surgical instrumentation consisted in the insertion of a catheter into the left carotid artery for monitoring systemic arterial blood pressure and for sampling arterial blood gases. General anesthesia was not used in order to avoid its effects on heart-rate and respiratory-rate variability (Venet et al., 2018). Antibiotics (ampicillin 50 mg and tobramycin 5 mg/kg) were also injected intramuscularly before anesthesia and once a day throughout the study.

The instrumentation was completed after a postoperative recovery period of 24 h, immediately before the experiments with: (i) subcutaneous needle electrodes for electroencephalogram (EEG), electrooculogram (EOG), and electrocardiogram (ECG) recordings; (ii) elastic bands on the chest and abdomen to record lung-volume variations semiquantitatively via respiratory inductance plethysmography; (iii) a pulse-oximeter probe placed at the base of the tail for oxygen hemoglobin saturation (SpO_2_); and (iv) a rectal probe to record body core temperature. Physiological signals were transmitted wirelessly using our custom-designed radiotelemetry system (Samson et al., 2011) and continuously recorded on a PC using the AcqKnowledge software (version 4.1, Biopac Systems, Montreal, QC, Canada).

### Design of the Study

All lambs were housed in a Plexiglas chamber (1.2 m long × 1.2 m wide × 1 m high with a floor surface of 1.44 m^2^) in accordance with the Canadian Council on Animal Care standards for housing one or two newborn lambs at the same time. They were able to move about and feed *ad libitum* from a custom-built lamb feeder (Duvareille et al., 2010). As previously described (Porée et al., 2014), an infrared video camera was positioned above the Plexiglas chamber to continuously monitor locomotor activity throughout the experiment.

#### Main Objective of the Study

##### Neonatal ovine model of systemic inflammation induced by lipopolysaccharide injection

Systemic inflammation was induced in eight full-term lambs weighing 3.3 ± 0.7 kg (range: 2.3–4.2 kg) by an intravenous injection of *Escherichia coli* lipopolysaccharides (LPS, 0127: B8, Sigma-Aldrich, St. Louis, MO, United States), a classical Toll-like receptor 4 (TLR4) agonist mostly involved in gram-negative bacterial infections.

After a postoperative recovery period of 24 h, two 6-h polysomnographic recordings were taken on non-sedated lambs on two consecutive mornings. During the first experimental day, each lamb received an intravenous bolus of 10 mL of normal saline solution (control condition, CTRL), whereas 10 mL of LPS from *E. coli* (2.5 μg/kg) were administered on the second day (LPS condition). An experimenter was present throughout the recording sessions. Arterial blood gases (pH, PaCO_2_, PaO_2_, and HCO_3_^–^) were measured at baseline and at time 3 and 6 h. Once the recordings had been completed, the subjects were euthanized with an IV injection of 90 mg/kg of pentobarbital sodium.

##### Video analysis of locomotor activity

The activity index, the total distance traveled, and the percentage of time the animal was active throughout the recordings were calculated with a custom software. The infrared video camera located above the Plexiglas chamber gave black-and-white top views of the scene with a resolution of 320 × 240 pixels at 30 fps. The software we developed to process the videos and extract the lamb’s trajectory is adapted to any static (or very slow varying) environment. Initialization of the image-processing algorithm was performed on the first frame of the video file by selecting the 100 best feature points with a standard Harris corner detector (Shi and Tomasi, 1994) (see details in Supplementary Figure 1). Thereafter, the feature points were tracked on the successive images with a Kanade–Lucas–Tomasi tracker (Tomasi and Kanade, 1991). At every iteration, the movement of each feature point above a threshold was computed to determine whether each point had moved between the two successive images. If the number of moving points was smaller than eight, the movement was considered as noise, e.g., limb motions while the lamb was lying. Conversely, a number of moving points greater than eight was taken as displacement of the lamb. Lastly, the lamb’s position in the image was computed by calculating the barycenter of the moving points and using the camera parameters (prior calibrations) and cage dimensions. The algorithm was coded in C++ with OpenCV library and used under a Windows 7 environment on an Intel^®^ Core^TM^ i7-5600U 2.6 GHz CPU.

##### States of alertness

Standard electrophysiological and behavioral criteria were used to define quiet and active wakefulness, as well as non-rapid eye movement (NREM) and rapid eye movement (REM) sleep (Renolleau et al., 1999).

##### Cardiac and respiratory function

Respiratory movements, ECG, and arterial blood pressure were continuously recorded for a period of 6 h during both control and LPS conditions. The following variables were calculated every 15 min: (i) f_R_, HR, and mean systemic arterial pressure (MAP) averaged on 30 s; (ii) the number of apneas (defined as at least two missed breaths) and the total apnea duration during the whole 6-h recording; (iii) the number of cardiac decelerations (defined as a decrease in HR greater than 30% lasting less than 5 s); and (iv) the number of bradycardias (defined as cardiac decelerations lasting at least 5 s).

##### Heart-rate and respiratory-rate variability

Our semiautomated processing approach, previously developed using Matlab R2013a software (The MathWorks Inc., Natick, MA, United States), was applied on the ECG and respiratory signals (Al-Omar et al., 2018). Each 6-h recording was automatically processed to extract all the 2-min stationary periods, which were considered to reflect the changes in autonomic state, according to previously proposed criteria (Borgnat et al., 2010). QRS complexes were then automatically detected as the maximum above a manually fixed threshold. The quality of each RR (cardiac-cycle length) series obtained was checked manually and corrected when necessary.

###### Heart-rate variability

Heart-rate variability reflects the heart’s adaptation to internal and external stimuli and is measured by the variation in RR intervals from the ECG signal. Analysis methods of RR interval time series were chosen considering past results on neonatal sepsis (Nguyen et al., 2017). Time-domain analysis of HRV included the mean and standard deviation (SD) of RR duration, an index of global HRV, and the square root of the mean squared differences of successive RR intervals (rMSSD), mainly reflecting parasympathetic control. The complexity and regularity of the RR series were assessed by computing the sample entropy (SampEn). The RR series, resampled at 4 Hz, were also subjected to frequency-domain analysis, through autoregressive estimation of the power spectrum and integration of the low-frequency (LF, 0.02–0.25 Hz, modulated by the arterial baroreflex) and high-frequency (HF, 0.25–2 Hz, from respiratory origin) spectral bands (Beuchée et al., 2007). The LF/HF ratio was calculated as an index of sympathovagal balance. In addition, the following non-linear analyses were performed. The Poincaré plot further assessed short-term (SD1) and long-term (SD2) HRV. Acceleration capacity (AC) and deceleration capacity (DC) were computed as previously described (Nguyen et al., 2017). The scale invariance (self-similarity of RR time series) was tested through the detrended fluctuation analysis technique, using the fractal scaling exponent α1 (from 4 to 40 beats) (Al-Omar et al., 2019).

In addition, the search for new methods with better accuracy to further improve the early detection of LOS led us to use HRV analyses based on the representations of the horizontal and vertical visibility networks (Lacasa et al., 2008; Madl, 2016; Nguyen Phuc Thu et al., 2019). Analysis of the horizontal and vertical visibility graphs allow for simultaneously assessing periodicity, fractality, and discontinuity properties of RR time series, hence providing novel global insight into HRV. The resulting dimensionless network representations (see Supplementary Figures 2–5, paragraph entitled “A Simple Introduction to Horizontal and Vertical Visibility Graphs” for a simplified, step-by-step explanation) are based on the organization of connectivity between the different durations of successive cardiac cycles. The networks derived from the visibility analyses have several interesting properties, such as differentiating stochastic, chaotic, and deterministic dynamical systems. Alterations in network graphic representations can be visually recognized and quantified by computing several variables, including the mean degree, assortativity, and transitivity (Luque et al., 2009).

###### Respiratory-rate variability

Respiratory-rate variability ensures respiratory-system stability. It results from the central respiratory drive issued from the brainstem respiratory centers, whose activity is modulated by multiple internal factors of nervous and chemical origin; some random variability reflects system sensitivity to external stimuli. Linear analyses of RRV in the time domain were performed, as previously described, on Ttot (total breathing cycle duration) series (Al-Omar et al., 2018) and included mean and SD computation. Non-linear analyses of Ttot variability included Poincaré plots (SD1 and SD2) and SampEn.

###### Cardiorespiratory interrelations

The Pearson *r*^2^ and the non-linear *h*_2_ correlation coefficients, the mean phase coherence γ_RR,RESP_, and the amplitude of the respiratory sinus arrhythmia (RSA, the difference between the maximum and the minimum RR in a respiratory cycle) (Carroll et al., 2012) were calculated, as previously detailed (Al-Omar et al., 2018).

#### Secondary Objective of the Study

##### Impact of LPS injection on the reflexivity of the autonomic nervous system

The laryngeal and pulmonary chemoreflexes were studied in a second group of eight full-term lambs weighing 4.1 ± 0.7 kg (range: 2.9–5.0 kg), 24 h after the surgical instrumentation described above. In addition, for the LCRs, a transcutaneous supraglottal catheter was inserted into the laryngeal vestibule, as previously described (Boudaa et al., 2013). Briefly, a 16-gauge infusion catheter was securely positioned such that its tip protruded 5–7.5 mm above the anterior part of the glottis. A plastic tube (internal diameter 1 mm) was subcutaneously tunneled in the neck of the animal and connected to the external part of the supraglottal catheter, which protruded 15–20 mm externally at the level of the anterosuperior aspect of the thyroid cartilage. The stimulations were performed once before LPS-injection and repeated 30 min, 2 and 24 h after LPS injection. The LCRs were induced with an injection of 0.5 mL HCl (pH 2) into the larynx during non-REM sleep. The PCRs were induced with an intravenous injection of 1 mL of capsaicin (10 μg/kg) into the jugular vein (Diaz et al., 1999). The occurrence of apneas, cardiac decelerations, and O_2_ hemoglobin desaturations was analyzed as described previously (Diaz et al., 1999; Boudaa et al., 2013).

##### Data analysis

Data collection was performed over 10 s immediately before each stimulation (baseline) and continued over the next 60-s period. The following inhibitory cardiorespiratory responses to each stimulation were computed: (1) the percent decrease in HR; (2) the number of cardiac decelerations (defined as a decrease in HR of at least 30% for <5 s); (3) the number of bradycardias (defined as cardiac deceleration lasting at least 5 s; (4) the total duration of cardiac inhibition (including cardiac decelerations and bradycardias); (5) the number and total duration of apneas (defined as at least two missed breaths), and (6) the percent decrease in O_2_ hemoglobin saturation.

#### Preliminary Studies on Brainstem Inflammation

##### Total RNA isolation, cDNA synthesis, and real-time quantitative PCR

Preliminary studies on brainstem inflammation were performed on four of the lambs from the main objective, 6 h after LPS injection, and on four additional control lambs, 6 h after saline injection. Following euthanasia, four control brains and four LPS brains were infused with cold PBS (1X). The brainstem was removed *en bloc* and kept in a TRIzol solution at –80°C. Total RNA extraction was performed using the RNeasy Midi Kit (Qiagen, Mississauga, ON, Canada) according to the manufacturer’s protocol. RNA concentration was measured with a Nanodrop 1000 (Thermo Fisher Scientific, Waltham, MA, United States) and RNA quality was measured with an Agilent 4200 Tapestation (Agilent, Mississauga, ON, Canada) to obtain the RNA integrity number. An amount of 1 μg of total RNA was reverse transcribed with the QuantiTect^®^ Reverse Transcription Kit (Qiagen) according to the manufacturer’s instructions. Quantitative PCR amplification was carried out for 40 cycles with the QuantStudio^TM^ 6 Flex Real−Time PCR System (Applied Biosystems, Foster City, CA, United States). Briefly, 1.5 μL of cDNA template were added to the master mix (TaqMan^®^ Universal PCR Master Mix, Thermo Fisher Scientific) with the appropriate primers. The amplification of IL-8 and TNF-α cDNA was carried out in triplicate on a 96-well plate in a final volume of 10 μL, including a negative water control for each primer. The qPCR conditions were as follows: 50°C for 2 min, 95°C for 10 min followed by 40 cycles of 95°C for 15 s and 60°C for 1 min. The cycle threshold values were automatically calculated using the manufacturer’s software. The delta-cycle threshold values were determined using the comparative CT method, as described previously (Schmittgen and Livak, 2008), and the relative quantification was normalized with the 2-ΔCT formula with the SDHA housekeeping gene as an endogenous control. The PCR probes and primers used are reported in Supplementary Table 1.

##### Tissue collection and immunofluorescent staining

Brainstems were dissected from the whole brains under saline perfusion, then fixed in 10% formalin solution, washed in PBS, and immerged in 30% sucrose solution at 4°C until they sank. The fixed brainstems were embedded in OCT (optimum cutting temperature) compound and frozen at −80°C. Sagittal sections 16 μm thick spaced at 160 μm were performed serially from the medial to the lateral brainstem using a Cryostat Thermo CryoStar NX50 (Thermo Fisher Scientific) and mounted on glass slides until use. Sections were warmed up at room temperature and equilibrated with PBS 1X, followed by an incubation period with a blocking solution containing 10% normal goat serum, 0.3% BSA, and 0.4% TritonX-100 for 30 min at room temperature. Subsequently, sections were incubated overnight at 4°C in a humidified chamber with these primary antibodies: rabbit polyclonal anti-Iba1 (1/400) (Wako, Richmond, VA, United States) and mouse monoclonal anti-GFAP (1/200) (New England Biolabs, Beverly, MA, United States). Brainstem sections were then washed three times with PBS 1X for 5 min and incubated with these secondary antibodies: goat anti-rabbit IgG AlexaFluor 488 (1:200) and goat anti-mouse IgG AlexaFluor 594 (1:500) (Life Technologies, Waltham, MA, United States) for 1 h at room temperature. After several washings, the slides were coverslipped with FluorSave^TM^ reagent (EMD Millipore, Billerica, MA, United States).

##### Analysis of microglial cell morphology

Microglial cells serve as resident macrophages in the nervous system and can be activated, e.g., by LPS, to produce various inflammatory mediators, such as IL-8 and TNF-α (Ehrlich et al., 1998; Welser-Alves and Milner, 2013). Upon activation, quiescent ramified microglia proliferate and transform into reactive microglia. For this proof-of-concept study, we compared the results from a total of six images (three sections × two images per section) obtained in one LPS-injected and one control lamb. Iba1-positive cells (microglia) of the rostral ventrolateral medulla were imaged using a Leica TCS SP8 confocal microscope (Leica, Deerfield, IL, United States) with a 63X objective generating 12.5–17.5 μm thick Z-stacks with a step size of 0.5 μm, a 1024 × 1024 μm image size, and a X-Y scale of 0.18 μm. The images were preprocessed to subtract background and remove speckles with ImageJ. Following qualitative assessment of the number and morphology of microglial cells, a quantitative 3D-morphological analysis of microglial cells using the 3DMorph Automatic Analysis Software, a MATLAB-based script (MathWorks, Natick, MA, United States) was performed. Output data of this analysis included individual cell volume, total territorial volume, and branch length (York et al., 2018).

#### Statistical Analysis

All statistical analyses were performed with help from our research center’s biostatistics department. Values were expressed as median (Q1, Q3). Statistical analyses were performed on raw data for all dependent variables. The Wilcoxon signed rank test was used to assess the effect of LPS injection on body core temperature, locomotor activity, states of alertness, apnea number, total apnea duration, cardiac deceleration, bradycardia number, and total cardiac deceleration duration. A similar analysis was also performed on the dependent variables computed for HRV, RRV and cardiorespiratory interrelations. To assess the effect of LPS on laryngeal and pulmonary chemoreflexes, a Friedman’s test was performed, followed by a *post hoc* Wilcoxon signed-rank test, when appropriate. All analyses were performed with SPSS Statistics for Windows (version 25, Armonk, NY, United States).

In addition, mixed models were used to evaluate the association between independent variables (recording-time point, temperature, and LPS injection) and each outcome variable (respiratory frequency, heart rate, and mean arterial pressure). Since multiple measurements were made in each lamb, a random effect on intercept and a first-order autoregressive structure of the residuals were specified in the covariance structure of the mixed models. Linear, quadratic, and cubic associations were studied for time and temperature, considering each day separately, and included in the multivariable models if significant. Models were obtained from PROC MIXED in SAS version 9.3 (SAS Institute Inc., Cary, NC, United States). Differences were considered significant if *p* < 0.05.

## Results

### Main Objective of the Study

#### Body Core Temperature

**FIGURE 1:**
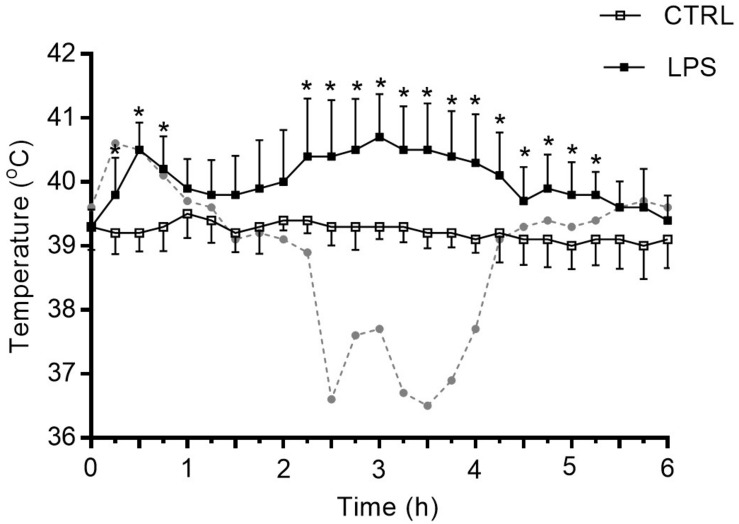
Variation of body core temperature after intravenous injection of lipopolysaccharide. LPS induced a biphasic increase in body core temperature in 7/8 newborn lambs. ▲: peaks of temperature. The dotted curve in gray represents the sole lamb who developed marked hypothermia following an initial increase in temperature. Results are illustrated as mean ± SD, *p < 0.05 vs. control condition (CTRL).

Contrarily to CTRL, LPS injection induced a biphasic increase in body core temperature in 7/8 lambs with a first peak at approximately 30 min (ΔT = 1.3°C; *p* = 0.01) followed by a second, longer increase peaking around 3 h (ΔT = 1.3°C; *p* = 0.01). Thereafter, body core temperature gradually decreased back to baseline level 5.5 h after LPS injection. Of note, one lamb out of eight presented marked hypothermia (max. ΔT −3°C around the 3-h time point) after the first temperature peak at 30 min (Figure 1). In addition to the changes in body temperature, all lambs were less active and presented diarrhea.

#### Locomotor Activity

**FIGURE 2:**
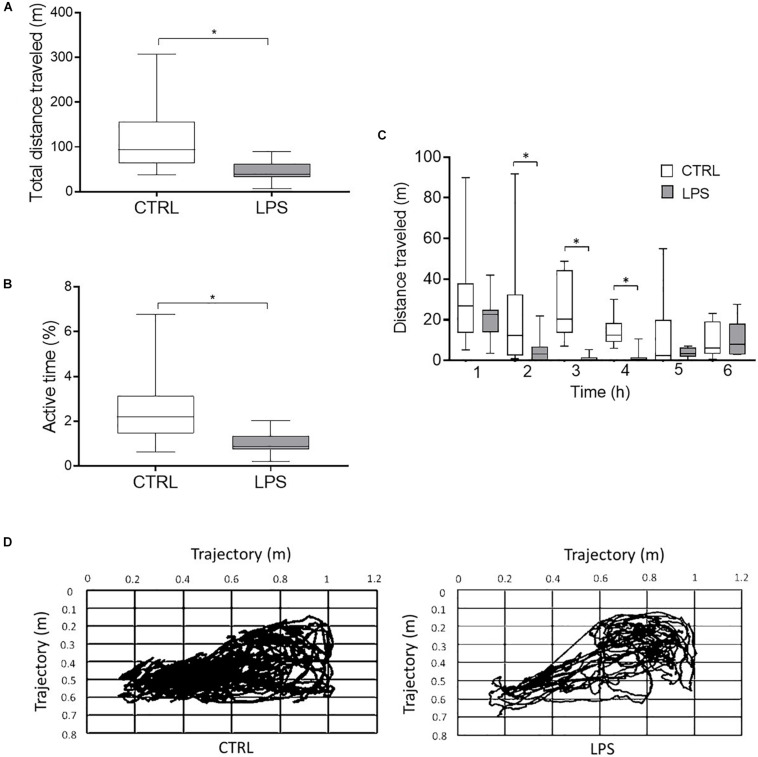
Video analysis of locomotor activity during the 6-h recordings. The total distance traveled **(A)** and the percentage of time the animal was active **(B)** were significantly decreased during the LPS compared to the control condition. In addition, the distance traveled per hour of recording **(C)** was significantly lower at 2, 3, and 4 h after LPS injection. Example of trajectory plots in the control and LPS conditions in one lamb **(D)**. Results are illustrated as median (Q1, Q3), *p < 0.05 vs. control condition (CTRL).

Video analysis (*n* = 7) revealed that LPS injection decreased the total distance traveled [38.7 (35.4, 54) vs. 93.8 (73, 128.1) m, *p* = 0.03] (Figures 2A,D) and the percentage of time the animal was active [0.9% (0.8, 1.2) vs. 2.2% (1.8, 2.8), *p* = 0.03] (Figure 2B) compared to the CTRL. Moreover, LPS injection significantly decreased the distance traveled per hour of recording at the 2-h [3.2 (0.4, 6) vs. 12.4 (4.5, 28.3) m, *p* = 0.04], 3-h [0 (0, 0.8) vs. 20.4 (15.6, 35.5) m, *p* = 0.02], and 4-h time points [0.7 (0.4, 1.5) vs. 12.5 (10.4, 16.6) m, *p* = 0.02] (Figure 2C).

#### States of Alertness

LPS injection significantly increased the percentage of recording time spent in quiet wakefulness [51% (47, 53) vs. 42% (37, 47), *p* = 0.03] and significantly decreased the percentage of time spent in active wakefulness [21% (17, 23) vs. 11% (7, 15), *p* = 0.01] compared to CTRL. No statistical differences were noted for NREM [28% (19, 31) vs. 21% (15, 25)] and REM sleep [5% (4, 6) vs. 5% (4, 9)] between the LPS and CTRLs, respectively.

#### Arterial Blood Gases

HCO_3_^–^ was significantly decreased at the 3-h [23 (22, 24) vs. 26 (25, 30) mmol/L, *p* = 0.03] and 6-h time points [25 (22, 26) vs. 30 (29, 31) mmol/L, *p* = 0.04] in the LPS compared to the CTRL, respectively. Similarly, PaCO_2_ was decreased at the 6-h time point [37 (30, 38) vs. 39 (36, 42) mmHg, *p* = 0.03] compared to the CTRL. No statistical differences were observed for pH and PaO_2_ between the two conditions (Table 1).

**TABLE 1:** Arterial blood gases for each condition.

	*T* = 0	*T* = 3	*T* = 6	*T* = 0	*T* = 3	*T* = 6
		
	Control *n* = 8			LPS *n* = 8		
PaO_2_ (mmHg)	79(73,93.3)	85(59,105)	93(72,96)	89(80,96)	110(98,126)	95(92,111)
PaCO_2_ (mmHg)	40(37,43)	36(31,42)	39(36,42)	39(37,41)	34(31,35)	37(30,38)*
pH	7.44(7.43,7.48)	7.49(7.48,7.5)	7.5(7.5,7.5)	7.45(7.43,7.47)	7.42(7.41,7.44)	7.46(7.44,7.48)
HCO_3_^–^ (mmol/L)	27(26,28)	26(25,30)	30(29,31)	27(25,29)	23(22,24)*	25(22,26)*

Results are expressed as median (Q1, Q3) at time 0 (*T* = 0), 3 h (*T* = 3), and 6 h (*T* = 6) after LPS injection. PaCO_2_ (partial pressure of carbon dioxide in arterial blood); PaO_2_ (partial pressure of oxygen in arterial blood); HCO_3_^–^, bicarbonate concentration in arterial blood. *p < 0.05: vs. control condition.

#### Effect of LPS Injection on Cardiorespiratory Control

##### Respiratory activity

**FIGURE 3:**
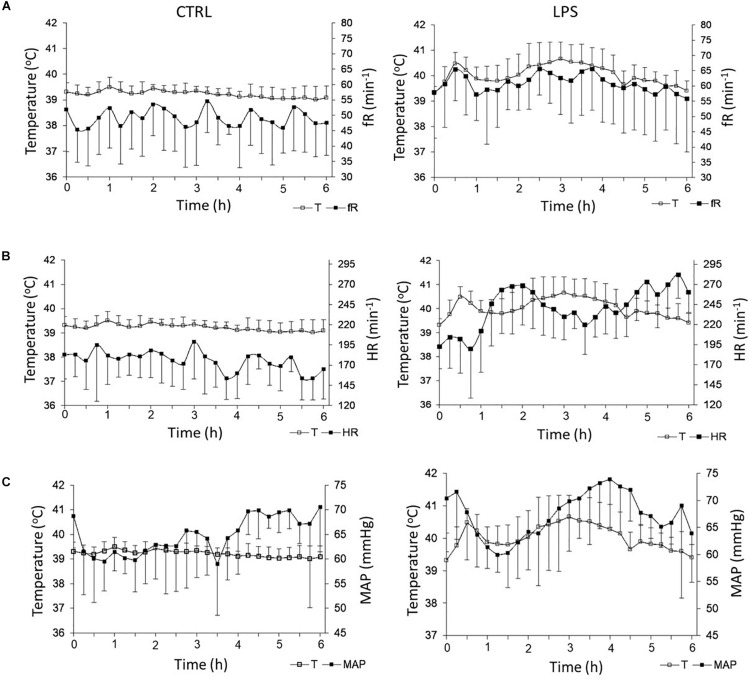
Effects of lipopolysaccharide injection on heart rate and respiratory frequency. LPS injection significantly increased respiratory frequency **(A)** and heart rate **(B)** compared to control condition. No significant alteration was observed for mean systemic arterial pressure **(C)**. Left panel: control condition (CTRL); right panel: LPS condition. f_R_, respiratory frequency; T, temperature; HR, heart rate; and MAP, mean arterial pressure. Results are illustrated as mean ± SD.

**FIGURE 4:**
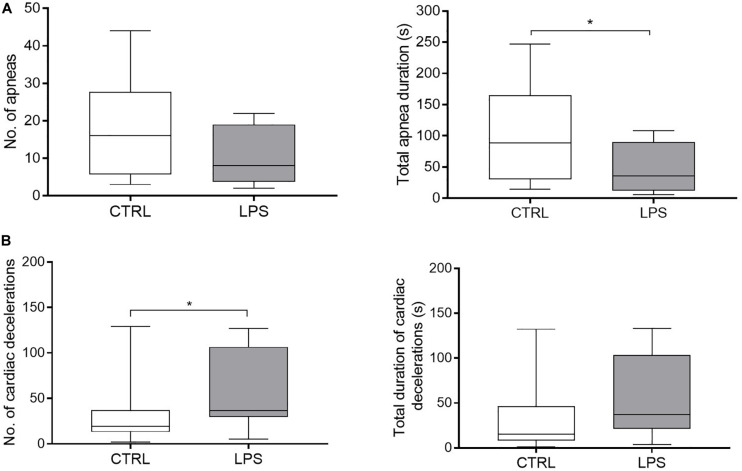
Effects of lipopolysaccharide injection on apnea and cardiac decelerations. **(A)** LPS injection significantly decreased total apnea duration compared to the control condition, but did not change the number of apneas. **(B)** LPS injection increased the number of cardiac decelerations, while no difference was observed for the total duration of cardiac decelerations. No, number. Results are illustrated as median (Q1, Q3), *p < 0.05 vs. control condition (CTRL).

Overall, LPS injection significantly increased f_R_ compared to the CTRL [61 (59, 63) vs. 49 (47, 52).min^–1^, *p* = 0.0005]. More precisely, f_R_ increased rapidly during the first 36 (30–40) min, then decreased until 60 min and rose again thereafter (Figure 3A). While the biphasic time course of f_R_ variations grossly reproduced that of temperature, no significant relationship was found between f_R_ and temperature. In addition, LPS injection significantly decreased total apnea duration compared to the CTRL [35.7 (18.2, 61.6) vs. 88.5 (42.7, 133.7) s, *p* = 0.04], while the number of apneas did not significantly decrease [8 (5.3, 12.3) vs. 16 (9.3, 21.8), *p* = 0.1] (Figure 4A).

The increase in f_R_ was confirmed when measured in the 2-min stationary periods for all lambs except one. Overall, the increase in the LPS condition exceeded 2 SD of the f_R_ observed in the CTRL after 30–40 min and was associated with a significant decrease in Ti/Ttot (ratio of inspiratory time over Ttot). The increase in f_R_ was associated with a significant decrease in RRV (SD of Ttot), which involved both short- and long-term variability, as measured by SD1 of Ttot and SD2 of Ttot of the Poincaré plot (Table 2).

**TABLE 2:** Heart-rate and respiratory-rate variability observed in stationary condition.

*N* = 8	Control	LPS at max1	*p*-value
RR (ms)	342(307,371)	237(219,258)	<10^–6^*
Ttot (ms)	1220(1066,1340)	921(818,984)	<10^–6^*
Ti (ms)	546(480,608)	386(353,409)	<10^–6^**
Te (ms)	647(538,732)	522(469,587)	<10^–6^*
Ti/Ttot	0.46(0.43,0.47)	0.43(0.41,0.44)	<10^–3^
**HRV**			
*SD* (ms)	9(7,13)	7(4,11)	NS
rMSSD (ms)	3(1,7)	2(1,5)	NS
SD1 (ms)	4(2,6)	3(1,5)	<10^–4^
SD2 (ms)	13(9,17)	9(5,15)	<10^–2^
HF (10^3^.ms^2^)	6.8(2.8,17.2)	3.3(1,11.3)	0.07
LF (10^3^.ms^2^)	26.1(12.9,45.2)	7.2(3,30.5)	<0.05
LF/HF	3.3(1.8,6.4)	2.6(1.8,4.3)	0.05
SampEn	0.19(0.07,0.39)	0.39(0.13,0.82)	NS
AC	0.30(0.04,0.69)	0.07(−0.02,0.25)	<10^–4^
DC	0.54(0.22,0.03)	0.14(0.05,0.38)	<10^–4^
α1	1.15(1.01,1.26)	1.15(1.05,1.28)	NS
V-MD	6.61(5.46,8.06)	5.37(4.51,6.88)	<10^–3^
V-assortativity	0.08(−0.01,0.13)	0.01(−0.03,0.06)	<10^–4^
V-transitivity	0.44(0.41,0.46)	0.42(0.40,0.44)	<10^–3^
H-MD	2.84(2.40,3.17)	2.81(2.04,3.10)	NS
H-assortativity	0.09(0.04,0.18)	0.04(0,0.09)	<10^–6^
H-transitivity	0.39(0.38,0.41)	0.36(0.35,0.37)	<10^–6^**
**RRV**			
*SD* Ttot (ms)	105(87,144)	60(49,83)	<10^–6^
SD1 Ttot (ms)	83(60,114)	49(39,72)	<10^–5^*
SD2 Ttot (ms)	125(94,178)	66(53,92)	<10^–6^
SampEn Ttot	2.3(2.14,2.72)	2.38(2.17,2.60)	NS
**Cardiorespiratory interrelations**			
*r*^2^	0.02(0.01,0.05)	0.02(0.01,0.05)	NS
*h*^2^	0.08(0.05,0.11)	0.08(0.05,0.25)	NS
γ_RR,RESP_ (n.u.)	0.08(0.04,0.11)	0.01(0.01,0.05)	<10^–5^
RSA (ms)	7.1(4.6,12.5)	5.8(3.4,9.2)	<0.05

Results are expressed as median (Q1, Q3). LPS at max1, variability computed at the time of the first peak in frequency after LPS injection. RR, cardiac-cycle length; Ttot, respiratory-cycle duration; Ti, inspiratory time; Te, expiratory time; SD, standard deviation; rMSSD, square root of the mean squared differences of successive RR intervals; SD1, Poincaré plot short-term variability coefficient; SD2, Poincaré plot long-term variability coefficient; HF, high frequency; LF, low frequency; SampEn, sample entropy; AC and DC, acceleration and deceleration capacities; α1, α1 fractal coefficient (detrended fluctuation analysis); *r*^2^, Pearson’s *r*^2^ correlation coefficient; *h*^2^, non-linear *h*^2^ correlation coefficient; γ_*RR*,*RESP*_, synchronization index; RSA, amplitude of respiratory sinus arrhythmia. The new variables used to describe vertical (V) and horizontal (H) visibility are the clustering coefficient [V-MD (vertical mean degree) and H-MD (horizontal mean degree)], the assortativity (V-assortativity and H-assortativity), and the transitivity (V-transitivity and H-transitivity). *p < 0.05 for all lambs except one; **p < 0.05 for all lambs.

##### Cardiac activity

Overall, LPS injection significantly increased HR compared to the CTRL [245 (229, 261) vs. 180 (172, 183).min^–1^, *p* = 0.005] (Figure 3B). Of note, conversely to f_R_, the time course of HR variations did not follow that of temperature. Indeed, although the increase in HR was also biphasic, the two phases were clearly delayed compared to f_R_ and temperature, the overall shape of the HR curve as a function of time mirroring that of both the f_R_ and temperature curves. When observed in the 2-min stationary periods, the change in HR expressed by the mean RR interval exceeded 2 SD of the HR observed in the CTRL after 63 (48–73) min and reached a first maximum at 115 (95–119) min, which corresponded to an amplitude of change of 5 SD. The second maximum was observed after more than 270 min. No significant relationship was found between HR and temperature.

Moreover, LPS injection significantly increased the number of cardiac decelerations (duration < 5 s) [37 (33, 89) vs. 19 (13, 34) *p* = 0.05] (Figure 4B). No bradycardia > 5 s was observed in either condition.

**FIGURE 5:**
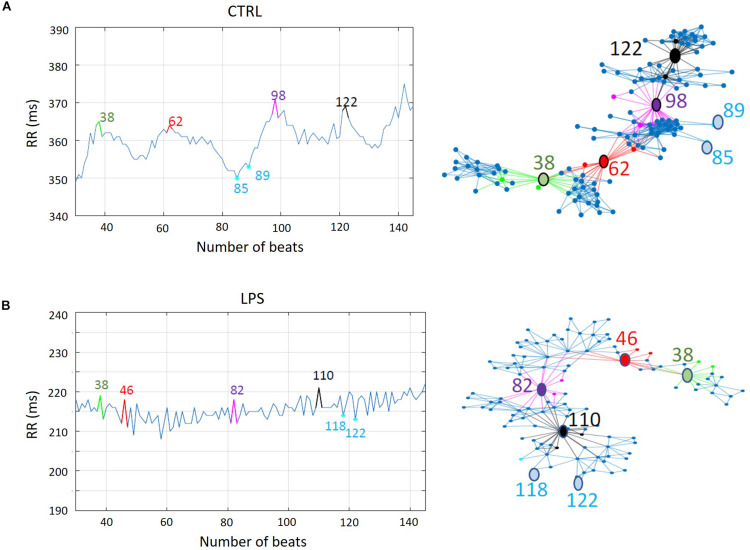
Impact of lipopolysaccharide injection on network representation of vertical visibility analysis of RR time series in one lamb. **(A)** The evolution of a RR time series (left) and the corresponding dimensionless network representation of the vertical visibility graph analysis computed with a horizon of 30 points (right). Some noteworthy nodes are annotated. For example, nodes 85 and 89, which are minima of the RR time series, have very few connections with the other nodes. Other nodes on the outer side are also minima. On the contrary, nodes 38, 62, 98, and 122–respectively in green, red, magenta, and black–are examples of maxima of the RR time series. The links (edges) in the same color show the multiple connections they have with the nearest neighbors. These maxima-related nodes are separated by subnetworks of highly connected nodes (in blue), whose size is related to the horizon distance. Note the overall high density of the links between nodes, which reflects the high cardiac variability in control condition. **(B)** A similar illustration following lipopolysaccharide injection in the same lamb. Similar to **(A)**, nodes 118 and 122—examples of minima of the RR time series—have very few connections with the other nodes. Contrary to **(A)** (representing the control condition), nodes 38, 46, 82, and 110—examples of maxima of the time series—are much less distinct nodes of the network. In addition, the subnetworks between these maxima-related nodes are barely identifiable, due to the fewer connections compared to the control condition. Such decreased connectivity attests to the low heart-rate variability, indicating an abnormal and inadequate adaptation of the autonomic nervous system. Of note, the orientation of the network representations has no particular meaning; it was only chosen to facilitate network interpretation.

The timing of changes in HRV were similar to the changes in HR (Table 2). The overall significant decrease in mean RR observed in all lambs was associated with a decrease in HRV, as measured by LF and HF (frequency-domain analysis), SD1 and SD2 (Poincaré plot), as well as AC and DC (non-linear analysis). Notably, results from the horizontal and vertical visibility analyses showed a highly significant decrease in most of the indices (mean degree, assortativity, and transitivity) computed from network representations (Table 2). Marked alterations in the organization of the latter were also readily apparent, as exemplified by the unequivocal decrease in connectivity and loss of well-defined hubs following LPS injection illustrated (see Figure 5).

##### Mean systemic arterial blood pressure

Overall, no significant difference was observed for MAP between the LPS and CTRLs [67.7 (63.9, 70.4) vs. 64.3 (61.5, 68.7) mmHg, *p* = 0.6]; moreover, there was no significant relationship between MAP and temperature (Figure 3C).

##### Cardiorespiratory interrelations

A decrease in both the phase synchronization and the magnitude of the respiratory sinus arrhythmia was observed, indicating a significant decrease in cardiorespiratory interactions (Table 2).

### Impact of LPS Injection on the Reflexivity of the Autonomic Nervous System

#### Laryngeal Chemoreflexes

No significant difference was observed for the cardiorespiratory components of the LCRs after LPS compared to saline injection, except for a significantly larger decrease in O_2_ hemoglobin saturation at 30 min compared to 24 h after LPS injection [2 (2, 3) vs. 1 (1, 2)%, *p* = 0.03] (Table 3).

**TABLE 3:** Absence of effects of LPS injection on the cardiorespiratory components of the laryngeal and pulmonary chemoreflexes in full-term lambs.

	Baseline		30 min post-LPS injection		2 h post-LPS injection		24 h post-LPS injection	
	LCR	PCR	LCR	PCR	LCR	PCR	LCR	PCR
No of. apneas	0(0,0)	1(1,1)	1(0,1)	2(1,2)	0(0,0)	1(1,2)	0(0,0)	1(1,1)
Total apnea duration, s	0(0,1,2)	4.8(3.3,8.2)	1.2(0,5.5)	7.8(6.4,15)	0(0,0)	7.3(3.3,16.5)	0(0,0)	7.9(4,9.3)
HR decrease,%	27(23,56)	73(78,70)	39(20,63)	81(38,88)	48(32,62)	86(77,89)	34(30,50)	78(70,82)
No. of cardiac decelerations	0(0,3)	8(5,8)	1(0,3)	5(1,8)	1(1,3)	12(6,13)	1(1,2)	8(5,11)
No. of bradycardias	0(0,0)	0(0,0)	0(0,0)	1(0,1)	0(0,0)	0(0,1)	0(0,0)	0(0,0)
Total cardiac inhibition duration, s	0(0,1.7)	5.1(4,11)	0.3(0,1.6)	7.9(4.9,16.2)	0.5(0.2,2.2)	7.7(6.2,22.1)	0.65(0.44,0.82)	7(5.8,9.9)
SpO_2_ decrease,%	1(1,1)	2(1,2)	2(2,3)‡		2(1,3)	4(2,5)*	1(1,2)	1(1,3)

LCR, laryngeal chemoreflexes; PCR, pulmonary chemoreflexes; HR, heart rate; SpO_2_, hemoglobin saturation in oxygen. Results are expressed as median (Q1, Q3) before LPS injection (baseline) and at 30 min, 2 and 24 h after LPS; n = 8 full-term lambs for each group. *p* < 0.05, * vs. baseline, ‡ vs. 24 h after LPS. The absence of SpO_2_ data 30 min after LPS injection is due to the fact that the oximetry sensor does not work when HR exceeds 300/min.

#### Pulmonary Chemoreflexes

No significant difference was observed for the cardiorespiratory components of the pulmonary chemoreflexes after LPS compared to saline injection, except for a significantly more important decrease in O_2_ hemoglobin saturation 2 h after LPS injection compared to baseline [4 (2, 5) vs. 2 (1, 2)%, *p* = 0.02] (Table 3).

### Preliminary Results on Brainstem Inflammation

#### IL-8 and TNF-α mRNA Expression

**FIGURE 6:**
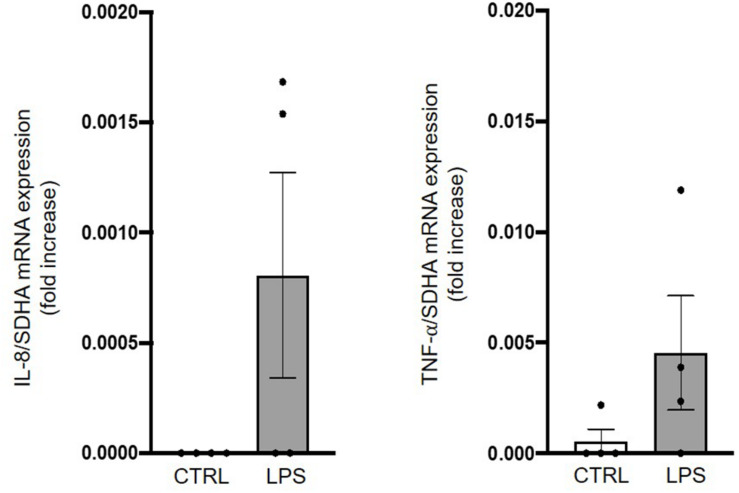
Assessment of brainstem inflammation following systemic lipopolysaccharide injection. Quantitative RT-PCRs in whole brainstem tissue show an increased IL-8 and TNF-α mRNA expression in LPS-injected lambs compared to control lambs. Results are expressed as fold increase compared to an endogenous control (the housekeeping gene SDHA) and presented as mean ± SD (*n* = 4 lambs per group).

A tendency toward an increase in mRNA expression of the early onset inflammatory mediators IL-8 and TNF-α was observed 6 h after LPS injection in four lambs compared to four controls (Figure 6).

#### Microglial Cell Activation in the Rostral Ventrolateral Medulla Region

**FIGURE 7:**
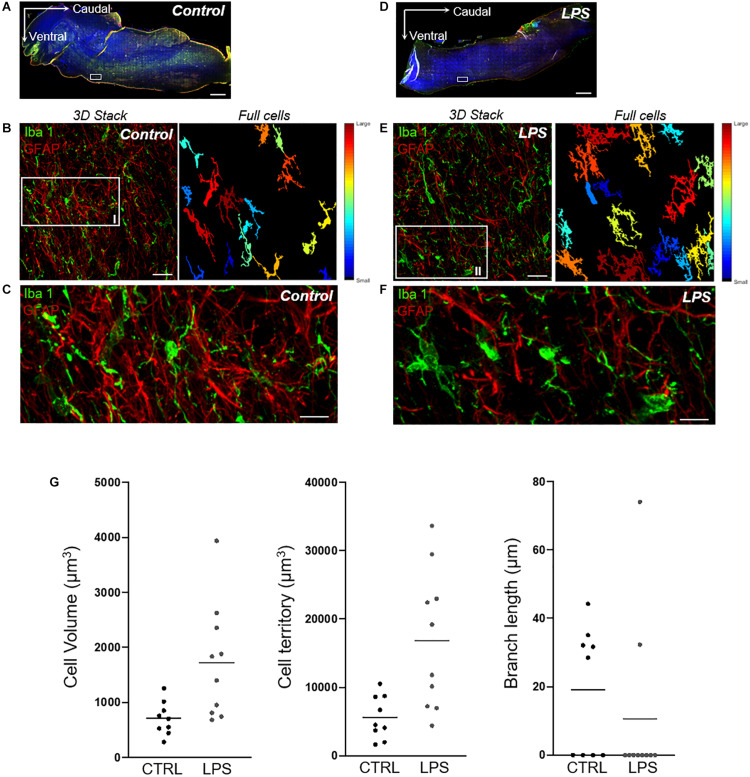
Quantitative analysis of microglial-cell morphology in the rostral ventrolateral region of the medulla. **(A,D)** Axioscan images of whole brainstem (AxioScan.Z1scanner, Carl Zeiss, Zaventem, Belgium) showing targeted areas from the rostral ventrolateral region of the medulla (white boxes) in one control (left) and one LPS-injected (right) lamb. Scale bar: 1000 μm. **(B,E)** Example of a 3D confocal image stack with full-cell segmentation automatically generated by 3DMorph MATLAB-based script showing how quantitative morphological analysis of microglial cells (Iba1-positive cells, green) is performed. The full-cell segmentation shows remaining microglial cells after exclusion of partial cells and small processes not related to a cell body. Scale bar: 35 μm. **(C,F)** High magnification of the same medullary areas allows for appreciation of the morphological changes of the activated microglial cells in the LPS-injected lamb compared to the resting state in the control lamb. Qualitative analysis thus reveals a higher number of microglial cells with a larger cell-body surface, a larger total territory occupied by microglial cells, and shorter branch length in the LPS-injected lamb. Meanwhile, qualitative assessment of astrogliosis (GFAP-positive cells, red) does not suggest differences between the LPS-injected and the control lamb over the same medullary regions. Scale bar: 10 μm. **(G)** 3DMorph quantitative analysis of microglial-cell morphology from the above images **(C,F)** shows an increased average cell volume and territorial volume, as well as smaller branch length in the LPS-injected compared to the control lamb.

Qualitative analyses performed in the medulla oblongata to assess morphological changes of microglial cells suggested an increased number of activated cells in the LPS-injected lamb compared to the control lamb. Meanwhile, the quantitative 3DMorph analysis of microglial cells on six images/lamb from the rostral ventrolateral medulla region showed a larger cell body volume [median (IQR): 1148 (1122) vs. 839 (825) μm^3^]; [mean ± SEM: 1425 ± 93 vs. 968 ± 79 μm^3^], a larger total territorial volume [8862 (11729) vs. 5267 (7629) μm^3^]; [11414 ± 876 vs. 7996 ± 983 μm^3^] occupied by microglial cells and fewer processes with shorter branch length [0 (27.9) vs. 16.5 (34.5) μm]; [14.8 ± 2.2 vs. 19.2 ± 3.0 μm] in the LPS-injected lamb compared to the control lamb (Figure 7).

## Discussion

This study provides new observations on the physiological alterations and their dynamics during the first 6 h of a systemic inflammation induced by LPS injection in a full-term newborn ovine model. Overall, we observed that the general effects of the LPS condition started within the first hour following the injection as a decrease in locomotor activity and active wakefulness as well as diarrhea and a biphasic increase in body core temperature. While the latter was mirrored by a simultaneous biphasic increase in respiratory frequency, the biphasic increase in HR was unexpectedly delayed. Moreover, an increase in the number of short cardiac decelerations was observed. Finally, LPS injection was followed by an overall decrease in both HRV and RRV, indicating an overall decrease in cardiac and respiratory regulatory capacity. Interestingly, the unique use of network analysis of HRV revealed especially marked alterations, suggesting such analysis might be of clinical importance for earlier recognition of neonatal sepsis.

### Our Neonatal Ovine Model of LPS-Induced Systemic Inflammation

An extensive review of the literature revealed that there are only a few reports on newborn ovine models of systemic inflammation using *E. coli* LPS (Billiards et al., 2002; McClure et al., 2005; Hamilton et al., 2006; Feng et al., 2010). In these reports, LPS doses ranging from 200 ng/kg to 3 μg/kg were administered, either as a bolus or infusion over 30 min. The 2.5 μg/kg bolus in our study was selected based on our pilot studies as the lowest dose of LPS that induced fever, decreased activity and diarrhea, as well as increased HR and f_R_ in lambs. This model hence mimics the systemic inflammation observed with bacterial sepsis without causing septic shock, as encountered, for instance, in the first hours of LOS in preterm newborns (Shane et al., 2017).

No previous studies on the effects of *E. coli* LPS in lambs (Billiards et al., 2002; McClure et al., 2005; Hamilton et al., 2006; Feng et al., 2010) specifically described alterations of cardiorespiratory control, apart from a brief mention of a marked increase in HR and respiratory frequency without any further details (Billiards et al., 2002). The systemic inflammation observed with bacterial sepsis was also mimicked by *E. coli* LPS injection in newborn rats (Olsson et al., 2003; McDonald et al., 2016a, b), mice (Mukherjee et al., 2010), piglets (McDeigan et al., 2003), and adult goats (Takeuchi et al., 1997). In neonatal rats, LPS injection tended to reduce f_R_ and the ability to autoresuscitate from an anoxic challenge (Olsson et al., 2003). Conversely, previous observations in newborn rats (McDonald et al., 2016a) and piglets (McDeigan et al., 2003) showed that LPS injection did not alter respiratory variables during normoxia. In neonatal mice, LPS injection increased HR and altered cardiac performance (Mukherjee et al., 2010), while, in adult sheep, LPS injection reduced MAP, HR, and cardiac index after 60 min (Ferrara et al., 2019). Finally, studies demonstrated that LPS injection tended to increase HR during the first 210 min in adult goats (Takeuchi et al., 1997) and significantly increased HR at higher ambient temperature (38°C) in rat pups (McDonald et al., 2016b). Overall, no clear picture emerged from past data, which might be related to the wide range of doses, the variable route of LPS administration, and ambient conditions. The LPS dose we used in lambs was selected to induce cardiorespiratory alterations during normoxia and in a neutral range of ambient temperature in order to mimic the systemic inflammation observed during the initial phase of bacterial sepsis without septic shock.

Our study provides unique observations, especially on cardiorespiratory alterations, in a neonatal model close to the full-term human newborn with bacterial sepsis. Although our full-term lamb model of systemic inflammation does not yet represent the preterm newborn with bacterial sepsis, our observations pave the way for further studies in our unique chronic preterm lamb model (Boudaa et al., 2013).

### Effect of Lipopolysaccharide Injection on Body Core Temperature

The dose of LPS used (2.5 μg/kg) in our study induced a biphasic increase in rectal temperature, which is characteristic of the reaction to LPS injection in most animal species (Roth and Blatteis, 2014), including in lambs aged < 20 days (Billiards et al., 2002; McClure et al., 2005). Several mechanisms are involved in the complex pathogenesis of this biphasic fever. They include activation of both TLR4 and the complement cascade, which results in the production/release of the pro-inflammatory cytokines IL-1β, IL-6, and TNF-α (via activation of the NFκB-pathway) and of PGE_2_ (via cyclooxygenase and PGE synthase induction). Macrophages in the liver (Küpffer cells) are especially involved in the production of these endogenous pyrogens initially. The pyrogenic message is then conveyed to the preoptic-area neurons in the hypothalamus, raising, in turn, the set point of the thermoregulatory center, via both a vagal afferent and a humoral pathway, in part explaining the early and late febrile phases, respectively (Roth and Blatteis, 2014; McDonald et al., 2017). Fever accelerates metabolism, promotes healing, and appears to inhibit bacterial growth. Thereby, this elevation in body temperature in our lamb model appears to be an appropriate central inflammatory response to LPS (Blomqvist and Engblom, 2018). Of note, moderate fever (temperature > 38°C) is a common though inconsistent clinical sign in human newborns with sepsis (Kerste et al., 2016; Wu et al., 2017).

### Effect of Lipopolysaccharide Injection on Locomotor Activity and Sleep States

Limited data are available on LPS-induced clinical manifestations in newborn animals. In our study, LPS injection decreased lamb locomotor activity compared to the CTRL, as reported previously (Roth and Blatteis, 2014). The proposed mechanism behind this reduction in locomotor activity is the involvement of pro-inflammatory cytokines, which act on the brain during infection in order to induce a sickness behavioral response characterized by drowsiness, loss of appetite, and decreased activity (Norheim et al., 2011). Although the increase in NREM sleep duration in the LPS condition compared to the CTRL did not reach significance in this study on lambs, quiet wakefulness was increased at the expense of active wakefulness in the LPS condition. This is consistent with a past report of an increase in drowsiness and NREM sleep in lambs after LPS injection (Billiards et al., 2002). The latter similarly increased NREM sleep in rodents and rabbits, while experimental results in healthy adult humans depended on the dose used, with NREM sleep being suppressed at high dose but increased at mild dose (Mullington et al., 2000). Alterations in alertness states, especially NREM sleep, are mediated by the action of cytokines on both individual sleep neuronal circuits and the sleep regulatory centers, such as the basal forebrain and preoptic area (Kapsimalis et al., 2005). Systemic cytokines affect brain function either via vagal afferent messages or by being transported across the blood–brain barrier. Central mechanisms of action involve neurotransmitters such as NO, adenosine, and glutamate receptor trafficking (Krueger and Opp, 2016). Of note, prostaglandins are also known to be involved in sleep regulation (Krueger and Opp, 2016).

### Effect of Lipopolysaccharide Injection on Cardiorespiratory Control

The main focus of our study was to investigate the effects of LPS injection on neonatal cardiorespiratory control. To our knowledge, this represents the first time that cardiorespiratory activity has been serially analyzed for several hours after LPS injection in a newborn animal. Overall, results showed an increase in both HR and f_R_ with no consistent changes in MAP. Interestingly, tachypnea, tachycardia, and HRV alterations are well-known signs of early- or late-onset sepsis in newborn humans (Griffin et al., 2005; Beuchée et al., 2009; Fairchild, 2013; Bohanon et al., 2015; Kerste et al., 2016; Nguyen et al., 2017; Wu et al., 2017).

#### Respiratory Activity

The biphasic increase in f_R_ we observed in lambs occurred early after LPS injection, was coincidental to the biphasic increase in temperature, and was associated with a decrease in RRV. Consequently, while a mild acute lung injury directly induced by intravenous LPS might have contributed to this increase (Wang et al., 2008), the in-phase increase in both f_R_ and temperature rather suggests a central effect of LPS-induced inflammation. It appears difficult, however, to attribute this to PGE_2_, which has been reported to inhibit respiratory activity, in particular by its effects on the prostaglandin EP3 receptors of neurons of the ventral respiratory column (Hofstetter et al., 2007; Siljehav et al., 2015; Forsberg et al., 2017). Of note, in contrast to our current results, intravenous LPS was reported to cause no respiratory alteration in neonatal rat pups (McDonald et al., 2016a, b) and piglets (McDeigan et al., 2003), or a small (insignificant) decrease in f_R_ in neonatal rat pups (Olsson et al., 2003). Discrepant results between those publications and our current study might be due to a number of methodological differences, including the LPS dose and administration route, timing of the measurement after LPS injection, and differences in species and age of the newborn animals.

#### Cardiac Activity

The increase in HR observed after LPS injection is consistent with past results in neonatal models (Mukherjee et al., 2010; McDonald et al., 2016a, b). It is ascribed to the LPS-induced increase in pro-inflammatory cytokines (e.g., IL-6, IL-1β, TNF-α) (Kenney and Ganta, 2014; Badke et al., 2018), which have a sympathoexcitatory effect. The latter is partly mediated by prostaglandin E_2_ acting on the prostaglandin EP3 receptors present at multiple sites in the central nervous system, including in the paraventricular nucleus of the hypothalamus, which signals the ventrolateral medulla and downstream sympathetic pathways (Kenney and Ganta, 2014).

Our observation of highly significant and early alterations in HRV following LPS injection is consistent with current pathophysiological knowledge. It can be interpreted as an early temporal disorganization of HR with a loss of adaptability due to an altered structure of the control system (Griffin et al., 2005; Beuchée et al., 2009; Madl, 2016). In this respect, it is worth mentioning that ours is the first report of a marked decrease in the indices that quantify the connectivity of the network representing the visibility graph analysis of HRV in the context of systemic neonatal inflammation. A novel message from the current results in lambs is that this decrease in network connectivity indices appears to be more pronounced than the alterations in the indices computed with the linear and non-linear analyses of HRV used to date. Of note, we (GC, PP) have made similar observations in preterm infants with LOS (unpublished results). Visibility graph analyses might consequently provide an earlier and more sensitive means to diagnose LOS in preterm infants. The latter hypothesis currently constitutes most of the relevance for performing visibility graph analyses, although this remains to be substantiated in future clinical studies. Lastly, from a physiological standpoint, it must be recognized that, beyond the temporal disorganization of HR, we currently have no information on the detailed alterations in the HR control system, which could explain the decreased connectivity of the networks resulting from visibility graph analyses. Such explanations would need physiological studies aiming to assess the effect of manipulating the sympathetic and parasympathetic branches of the autonomic nervous system on the results of visibility graph analyses, as described for past HRV analyses (Draghici and Taylor, 2016).

The unique observation of the manifest dissociation between the biphasic increase in HR and temperature is intriguing. It is reminiscent of the relative bradycardia (or sphygmothermic dissociation) observed in some infectious conditions such as salmonella infections (Ye et al., 2018). This relative bradycardia is thought to result from the complex interactions existing between the immune system and the sympathetic and parasympathetic arms of the autonomic system in the presence of inflammation (Kenney and Ganta, 2014). A simplistic scheme would be that the increase in pro-inflammatory cytokines consequent to endotoxin injection triggers parasympathetic activation, which decreases HR. The latter, in turn, leads to sympathetic activation, which is then responsible for an increase in HR (Ye et al., 2018). Lastly, while reminiscent of the bradycardias specifically observed with neonatal infections (Beuchée et al., 2009), the overall increased number of cardiac decelerations after LPS injection compared to the CTRL might be due a central effect of PGE_2_. In this respect, PGE_2_ infusion has been shown to slow HR in fetal sheep (Savich et al., 1995). Of note, we did not find any evidence of increased parasympathetic activity to explain the cardiac decelerations.

### Absence of Effect of Lipopolysaccharide Injection on Cardiorespiratory Reflex Responses

Results from a past study by our team showed that the presence of brainstem inflammation induced by a moderate hyperbilirubinemia blunted the cardiorespiratory inhibition normally observed during both laryngeal and pulmonary chemoreflexes (Specq et al., 2016). The absence of any effect in the present experiments in full-term lambs, despite a preliminary indication (see next paragraph) that brainstem inflammation was also present, is probably be linked to the fact that the former study was performed on preterm lambs. Indeed, although a very significant cardiorespiratory inhibitory response was usually present in the latter, especially during the LCRs, this was not the case for full-term lambs, making the possibility to observe a decrease in this cardiorespiratory inhibition unlikely. In addition, there was no enhancement of this response, which would have also been a possibility in the context of the inflammatory reflex, in which efferent vagal activity is a major component (Tracey, 2002).

### Preliminary Results on Brainstem Inflammation

Although very preliminary, we believe that our results showing a trend for an increased mRNA expression of the early inflammatory markers IL-8 and TNF-α in the brainstem, as well as activation of microglial cells in the region of the rostral ventrolateral medulla, are significant. Indeed, they provide proof of principle that inflammation can be shown in the vicinity of important cardiorespiratory centers in LPS-injected newborn lambs. Such inflammation might, at least partly, explain the cardiorespiratory alterations that we report herein. Our preliminary results hence are in agreement and extend past reports on rodents that cytokines in the brainstem, either produced locally−especially by microglial cells−or accessing the brain via the circumventricular organs, can alter the neuronal function involved in respiratory control in early life, in part via PGE_2_ synthesis (Gresham et al., 2011; Stojanovska et al., 2018).

Although several explanations might account for the fact that an increase in IL-8 and TNF-α mRNA was not detected in all lambs 6 h after LPS injection, the interindividual variability of the time lag between LPS injection and the inflammatory response is the most likely. Past studies in fetal lambs reported that the highest serum level of IL-6 and TNF-α was measured between 3 and 6 h after systemic injection of LPS in fetal lambs (Durosier et al., 2015; Herry et al., 2016). Given the expectation that cytokine production and release by the nervous system will peak later on, our 6-h timepoint might be too early to observe a consistent increase in cytokine mRNA expression in the lambs’ brainstem.

### Study Limitations

The main goal of our study was to further characterize the cardiorespiratory alterations observed during several hours after LPS injection in full-term newborn lambs. Systemic injection of LPS, especially in mice, has been widely used as a preclinical model of sepsis. It has been shown, however, not to fully replicate the systemic inflammation observed during sepsis, whose onset is more progressive and duration longer. Although the effects of LPS injection in lambs appear closer to those observed in humans (e.g., for the dose needed to induce a significant systemic inflammation) their temporal characteristics are still different from those observed with sepsis (Zanotti-Cavazzoni and Goldfarb, 2009). Hence, our lamb model studied herein is mostly a model of acute, transient systemic inflammation.

While the tachycardia and tachypnea we observed in the lambs are recognized signs of neonatal sepsis in human newborns, we did not observe the severe apneas/bradycardias that can reveal systemic infection in the human infant during the very first weeks of life, especially in the preterm infant with LOS (Weber et al., 2008; Beuchée et al., 2009; Zuckerbraun et al., 2009). This was not unexpected, however, since cardiorespiratory events associated with sepsis are less frequent in full-term than preterm human newborns. In this respect, our results in full-term lambs must be seen as a first step paving the way for further studies in a preterm lamb model of systemic inflammation with cardiorespiratory control immaturity (Renolleau et al., 1999; St-Hilaire et al., 2007; Nault et al., 2017).

As already alluded to, while our preliminary results showing an increase in cytokine mRNA expression and microglial activity suggest the presence of brainstem inflammation in newborn lambs 6 h after LPS injection, they only provide a proof of concept. These preliminary observations must be considered as the very first step in studying inflammation in the vicinity of cardiorespiratory centers and will have to be rounded out with a greater number of analyses in lambs, especially preterms.

Lastly, given that neonatal sepsis can also result from a viral infection, we believe that further studies with a lamb model of systemic inflammation mimicking that induced by a viral infection would be relevant to characterize its effects on cardiorespiratory control. For example, this could involve IV injection of poly I:C—a classical TLR3 agonist involved in infections by rhinovirus, respiratory syncytial virus, and influenza virus. The results from such a study could be compared to our current results.

## Conclusion

Our study confirmed that LPS injection in full-term newborn lambs reproduces several features of the systemic inflammation observed in the early phase of bacterial sepsis, including cardiorespiratory control alterations. The latter are complex, as uniquely shown, for instance, by the simultaneous biphasic increase in temperature and respiratory frequency, contrasting with the unexplained delay in increased HR. In addition, we believe that the highly significant alterations in HR variability shown by visibility graph analyses uniquely applied to systemic inflammation are clinically relevant as a novel means to provide an earlier diagnosis of LOS in preterm human newborns. Further studies are needed in a preterm ovine model to better mimic the conditions encountered in preterm infants with LOS. In addition, studies in preterm lambs will have to thoroughly characterize the inflammatory profile in the brainstem cardiorespiratory centers in order to gain further insight into the mechanisms involved and ultimately provide better care to young infants with sepsis.

## Data Availability Statement

The datasets generated for this study are available on request to the corresponding author.

## Ethics Statement

The animal study was reviewed and approved by Ethics Committee for Animal Care and Experimentation of the Université de Sherbrooke. Written informed consent was obtained from the owners for the participation of their animals in this study.

## Author Contributions

J-PP, NS, PP, SN, ST, and VC conceived and designed the study. CN and SN performed the animal experiments. AL, GC, J-PP, RI, SA-O, SN, ST, and VC analyzed the data. GC, J-PP, PP, RI, SN, and ST prepared the figures. All the authors contributed to the manuscript draft, and revised, read, and approved the final version of the manuscript.

## Conflict of Interest

The authors declare that the research was conducted in the absence of any commercial or financial relationships that could be construed as a potential conflict of interest.

## Abbreviations

AC: acceleration capacity
CTRL: control condition
DC: deceleration capacity
*E. coli*: *Escherichia coli;*
ECG: electrocardiogram
EEG: electroencephalogram
EOG: electrooculogram
f_R_: respiratory frequency
HCO_3_^–^: bicarbonate concentration in arterial blood
HF: high-frequency
H-MD: horizontal mean degree
HR: heart rate
HRV: heart-rate variability
IL-8: interleukine 8
LCR: Laryngeal chemoreflex
LH: Low-frequency
LOS: late-onset sepsis
LPS: lipopolysaccharide
MAP: mean systemic arterial pressure
mRNA: messenger ribonucleic acid
NREM: non-rapid eye movement
PaCO_2_: partial pressure of carbon dioxide in arterial blood
PaO_2_: partial pressure of oxygen in arterial blood
PCR: pulmonary chemoreflex
PGE_2_: Prostaglandin E_2_
REM: Rapid eye movement
RMSSD: square root of the mean squared differences of successive RR intervals
RRV: respiratory-rate variability
RSA: respiratory sinus arrhythmia
SampEn: Sample entropy
SD1: short-term heart-rate variability
SD2: long-term heart-rate variability
SpO_2_: oxygen hemoglobin saturation
TNFα: tumor necrosis factor alpha
TLR4: toll-like receptor 4
V-MD: vertical mean degree.
