# Supplementary material for: Cardiorespiratory Alterations in a Newborn Ovine Model of Systemic Inflammation Induced by Lipopolysaccharide Injection
Source: Front Physiol. 2020 Jun 17;11:585. doi: 10.3389/fphys.2020.00585 (PMC7311791; doi:10.3389/fphys.2020.00585)

Supplementary Figure 1. Custom image-processing algorithm to detect lamb's position in the video frames.

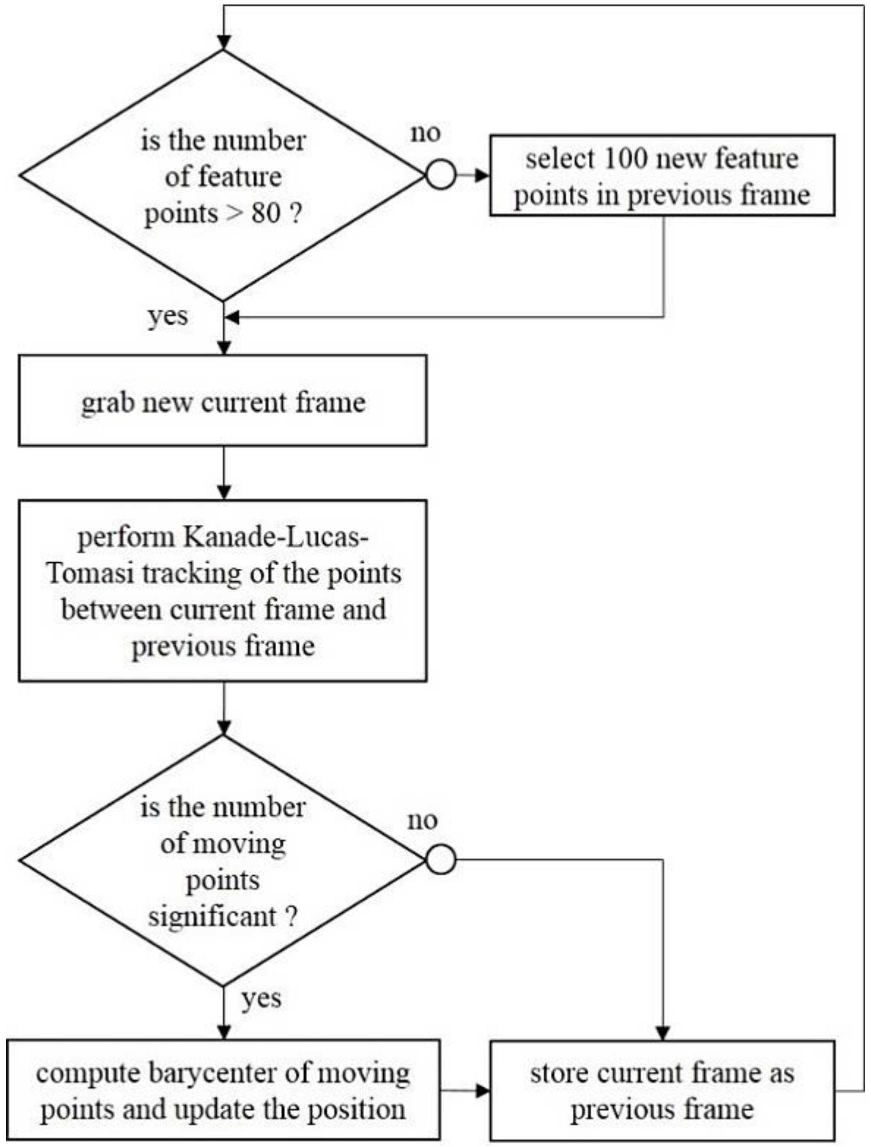

Supplement: Supplementary file 1 [file Data_Sheet_1.PDF]
