# Supplementary material for: Cardiorespiratory Alterations in a Newborn Ovine Model of Systemic Inflammation Induced by Lipopolysaccharide Injection
Source: Front Physiol. 2020 Jun 17;11:585. doi: 10.3389/fphys.2020.00585 (PMC7311791; doi:10.3389/fphys.2020.00585)
Supplement: Supplementary file 2 [file Data_Sheet_2.PDF]

A simple introduction to horizontal and vertical visibility graphs

Horizontal-visibility analysis

1. Suppose that we observed the following RR time series in which each point is characterized by a doublet (t_i, y_i), t_i being the ith beat and y_i the preceding RR interval.

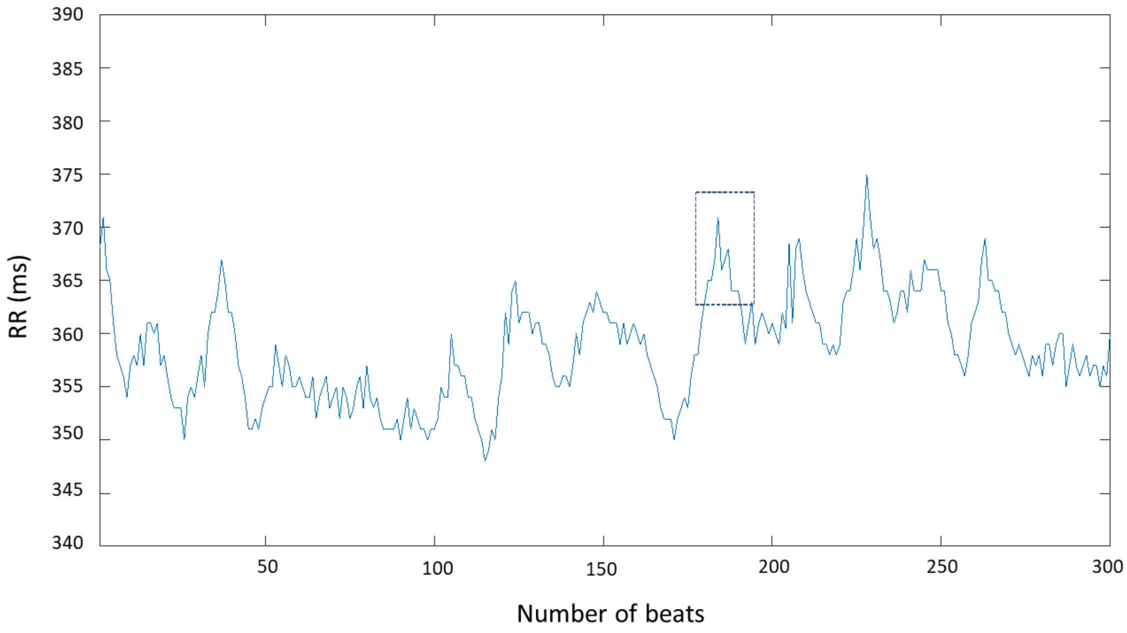

Supplementary Figure 2. Graphic representation of an RR series, in which the abscissa is the number of each beat in the time series and the ordinate is the value of the RR interval in milliseconds.

2. For simplicity, suppose that we take the excerpt in the box in Supplementary Figure 2, and convert the time-series values within this excerpt into an associated visibility graph that we consider as a landscape (Supplementary Figure 3). Two arbitrary data values of the time series (t_i, y_i) and (t_j, y_j) have visibility in this landscape if any other data (t_k, y_k) observed between them ($t_i < t_k < t_j$) fulfills the following visibility criterion:

$$\forall t_k \in [t_i, t_j]: y_i > y_k \; and \; y_j > y_k$$

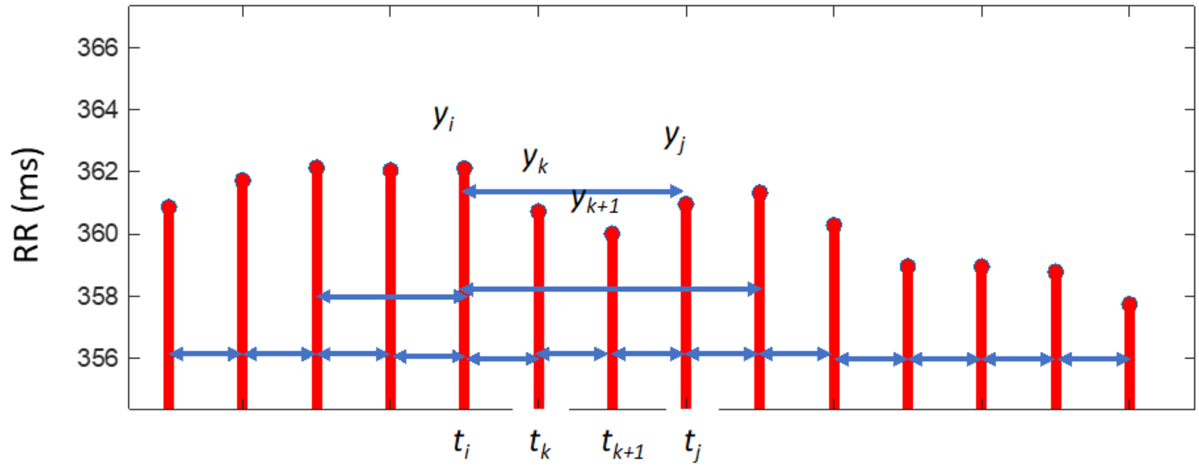

Supplementary Figure 3. Graphic horizontal visibility graph associated with the excerpt from Supplementary Figure 2.

3. Such analysis can be applied to the whole RR time series above using a 60-beat sliding window, for example. Hence, a network graph can finally be drawn to illustrate the overall visibility analysis (Supplementary Figure 4), in which every data point within the sliding window is transformed into a node and connected to visible nodes. In the present case, a link between two nodes is represented by a straight line (edge) in the whole time series.

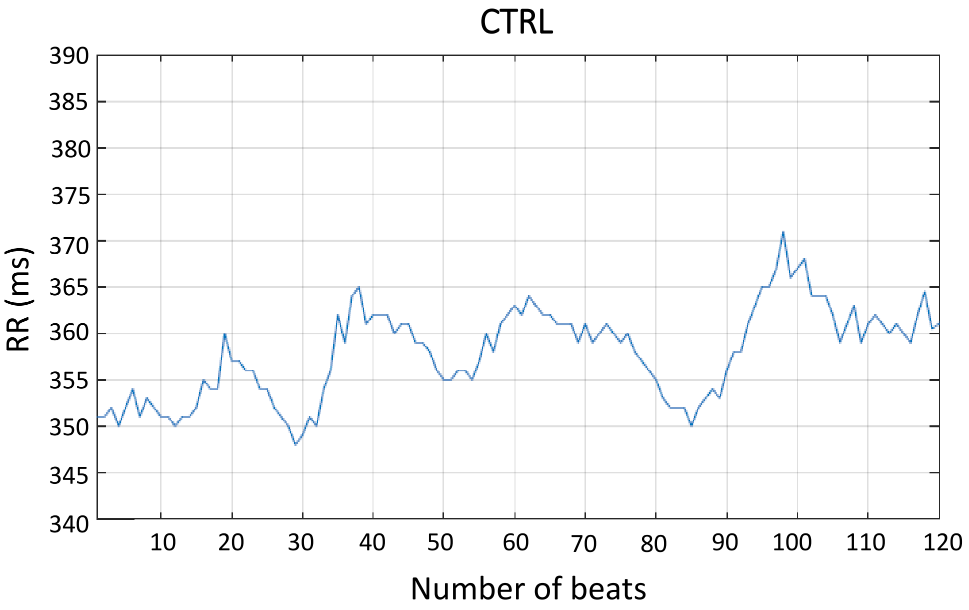

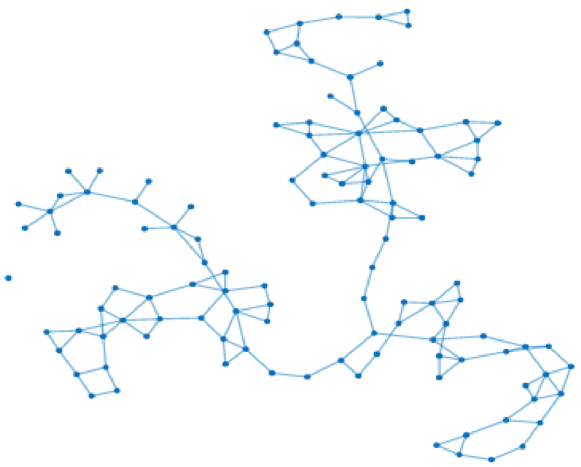

Horizontal visibility network from the CTRL time series

Supplementary Figure 4. Horizontal-visibility network graph illustrating the horizontal visibility analysis of the whole time series depicted in Supplementary Figure 1. This time series was obtained after saline injection (control) in one lamb.

4. From this network graph, we can compute the number of nodes and the number of edges arriving at a node y_i, i.e. the degree of this node. We can also compute the number of triangles (= closed triplets) and the total number of (open and closed) triplets. Three measures can then be retained using graph theory to characterize the visibility network graph:
    - The mean degree (H_MD) calculated on the entire graph. H_MD is a quantification of the network complexity. For example, in the present study the saline horizontal-visibility network graph is more complex than the LPS horizontal-visibility graph.
    - The H-assortativity which is an indirect measure of the connectivity of nodes with the same degree. Assortativity provides information about the structure of the network.
    - The H-transitivity which is the proportion of closed triangles among all possible triplets present in the graph. The H-transitivity quantifies the global connectivity within the network graph.
    -

Vertical visibility analysis:

1- An analysis similar to the one used for the horizontal visibility network graph is applied to construct the vertical visibility network graph. Two arbitrary data values of the time series (t_i, y_i) and (t_j, y_j) are visible and will become connected nodes if any other data (t_k, y_k) observed between them ($t_i < t_k < t_j$) fulfills the following visibility criterion:

$$y_k < y_j + (y_i - y_j)\frac{t_j - t_k}{t_j - t_i}$$

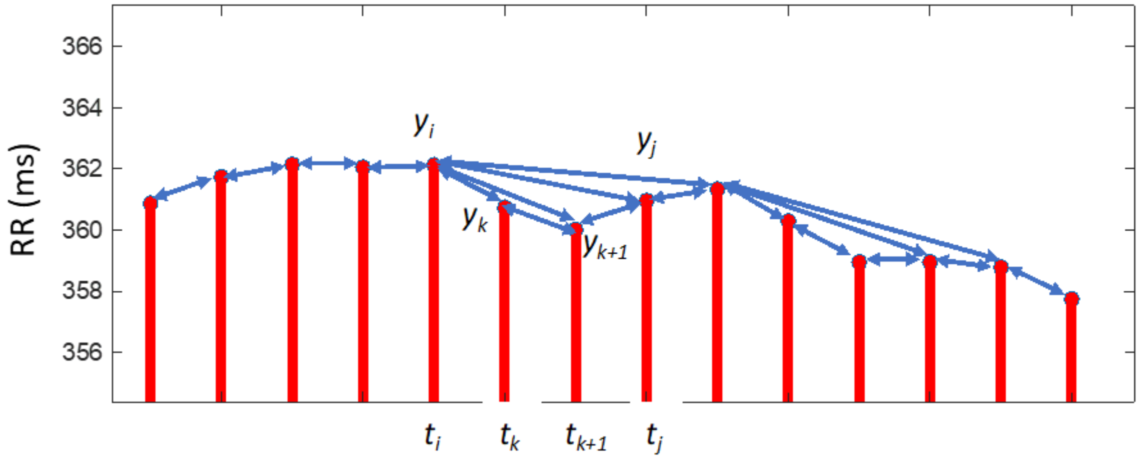

Supplementary Figure 5. Vertical visibility graph associated with the excerpt from Supplementary Figure 2.

2- The rest of the analysis is similar to that described for the horizontal-visibility analysis.
