# Supplementary material for: Cardiorespiratory Alterations in a Newborn Ovine Model of Systemic Inflammation Induced by Lipopolysaccharide Injection
Source: Front Physiol. 2020 Jun 17;11:585. doi: 10.3389/fphys.2020.00585 (PMC7311791; doi:10.3389/fphys.2020.00585)
Supplement: Supplementary file 3 [file Data_Sheet_3.PDF]

Supplementary Table 1. PCR probes and primers (Invitrogen) used for the study of brainstem inflammation

Gene Symbol	Ref-Seq	Dye-Label	Taqman Assay ID
SDHA	XM_027980212.1	FAM-MGB	Oa04307499_m1
CXCL8	NM_001009401.2	FAM-MGB	Oa04655586_m1
TNF-alpha	NM_001024860.1	FAM-MGB	Oa04656867_g1
